# Psychological impact and coping strategies of medical students during university closure due to COVID-19 in a state university in Sri Lanka – an online survey

**DOI:** 10.1192/j.eurpsy.2021.1751

**Published:** 2021-08-13

**Authors:** Y. Rohanachandra, P. Seneviratne, L. Amarakoon, S. Prathapan

**Affiliations:** 1 Department Of Psychiatry, University of Sri Jayewardenepura, Nugegoda, Sri Lanka; 2 Department Of Community Medicine, University of Sri Jayewardenepura, Nugegoda, Sri Lanka

**Keywords:** medical undergraduates, Depression, Anxiety, stress

## Abstract

**Introduction:**

Loss of routine, disengagement from peers and adapting to distant learning during the pandemic may lead to psychological distress in medical students. Psychological impact of the pandemic on medical students has not been assessed in Sri Lanka

**Objectives:**

To identify the psychological impact and coping strategies of medical students during the pandemic.

**Methods:**

An online survey was done among 527 medical students in a state Medical Faculty in Sri Lanka. Depression Anxiety Stress Scale (DASS-21) was used to measure psychological impact.

**Results:**

The main worries among the students was upcoming exams (74.4%, n=389) and taking a longer time than expected to complete their undergraduate medical education (68.1%, n=356). 68.7% (n=362) of the respondents experienced difficulty in working up the initiative to do things and 62.6% (n=330) had tendency to overreact to situations. Depressive symptoms were present in 40.8%, anxiety in 34% and high levels of stress were seen in 24.7%. In 10.8% depression was severe and anxiety was severe in 10.3%. Depression (p<0.01), anxiety (p<0.05) and stress (p<0.01) were significantly higher in students with a past history of psychiatric disorders. Depression, anxiety or stress was not associated with the gender, ethnicity, family income or living circumstances. The main coping strategy of the participants was engaging more with family (73.4%).

**Conclusions:**

Given the high levels of psychological distress, supportive strategies should be designed to minimize the psychological impact in this vulnerable group.

**Disclosure:**

No significant relationships.

